# Supplementary material for: Cumulative Weighing of Time in Intertemporal Tradeoffs
Source: J Exp Psychol Gen. 2016 Sep;145(9):1177–205. doi: 10.1037/xge0000198 (PMC4998108; doi:10.1037/xge0000198)
Supplement: Supplementary file 1 [file Scholten_Individuals.zip › plots/e30_p586_eg2_priors_and_posteriors.pdf]

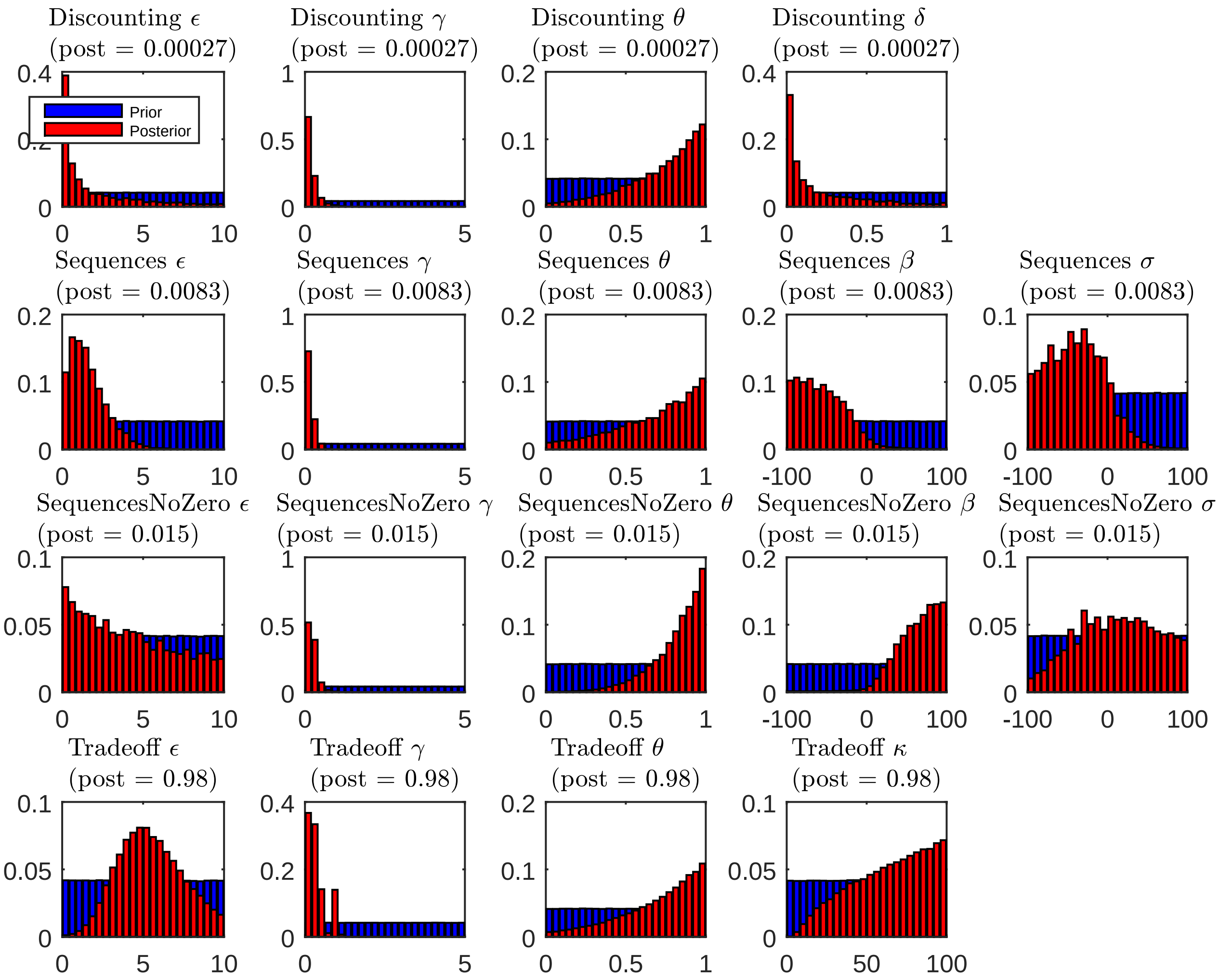
Discounting ϵ (post = 0.00027)
Discounting γ (post = 0.00027)
Discounting θ (post = 0.00027)
Discounting δ (post = 0.00027)
Prior
Posterior
Sequences ϵ (post = 0.0083)
Sequences γ (post = 0.0083)
Sequences θ (post = 0.0083)
Sequences β (post = 0.0083)
Sequences σ (post = 0.0083)
SequencesNoZero ϵ (post = 0.015)
SequencesNoZero γ (post = 0.015)
SequencesNoZero θ (post = 0.015)
SequencesNoZero β (post = 0.015)
SequencesNoZero σ (post = 0.015)
Tradeoff ϵ (post = 0.98)
Tradeoff γ (post = 0.98)
Tradeoff θ (post = 0.98)
Tradeoff κ (post = 0.98)
